# The Austrian Spinal Cord Injury Study: a registry for patients living with a traumatic spinal cord injury

**DOI:** 10.1038/s41394-017-0006-y

**Published:** 2017-10-23

**Authors:** Stephanie Aschauer-Wallner, Georg Mattiassich, Ludwig Aigner, Herbert Resch, on behalf of the ASCIS study group

**Affiliations:** 1 Department of Orthopedics and Traumatology, Spinal Cord Injury and Tissue Regeneration Center Salzburg, Paracelsus Medical University Salzburg, Salzburg, Austria; 2 Trauma Center Linz, Teaching Hospital of the Paracelsus Medical University Salzburg, Linz, Austria; 3 Ludwig-Boltzmann Institute for Experimental and Clinical Traumatology, Vienna, Austria; 4 Department of Orthopedics, Ordensklinikum Linz Barmherzige Schwestern, Linz, Austria; 5 Spinal Cord Injury and Tissue Regeneration Center Salzburg, Institute of Molecular Regenerative Medicine, Paracelsus Medical University, Salzburg, Austria

## Abstract

**Study design:**

Establishing the structure of a prospective spinal cord injury (SCI) patient registry.

**Objectives:**

To develop a registry for patients with traumatic spinal cord injury (tSCI) in Austria as a base for addressing research questions, improving patient outcomes, and establishing a platform for future clinical trials.

**Settings:**

Coordinating institution: Paracelsus Medical University Salzburg, Austria; participating partners are located in nine states in Austria.

**Methods:**

The Austrian Spinal Cord Injury Study (ASCIS) collects longitudinal data on simple forms within a 7-stage follow-up examination timeline.

**Results:**

The implementation of the ASCIS in 2012 created the first nationwide SCI patient registry in Austria. ASCIS is currently implemented in 17 trauma hospitals in 9 Austrian states, and over 150 individuals with acute tSCI have been registered to date. As in Austria, the structure of the health-care system does not involve a specialized SCI center covering the primary health care and the rehabilitation care, major challenges have to be overcome to involve all participating primary centers and rehabilitation centers, which perform tSCI patient care, for ASCIS. Through implementing ASCIS, a network of SCI clinicians and researchers, which is now beginning to support translational research and to initiate clinical trials for patients with tSCI, has formed.

**Conclusions:**

ASCIS is uniquely positioned in Austria to capture detailed information from the early acute to the chronic phases of tSCI, to provide this information also to bigger and translational settings, and to connect researchers and clinicians to facilitate clinical research on tSCI.

## Introduction

Traumatic spinal cord injury (tSCI) is a debilitating disease that leads to neurological deficits and often has long-term effects including severe life-long disability. In Western countries, tSCI affects 15–53 new individuals per million people each year and presents a considerable burden to the health-care system [1, 2]. Individuals who sustain a spinal cord injury (SCI) frequently require assistance with personal care and activities of daily living in addition to experiencing medical complications such as neurogenic bladder and bowel dysfunction, spasticity, and pain [3–5]. SCI thus results in significant initial and ongoing health-care costs, which may escalate over time with advancing medical technology and the increasing life expectancies of this population [6, 7]. Given the devastating personal and economic consequences of SCI, it is critical to collect high-quality, prospective data. However, SCI has not been systemically assessed in Austria, and data regarding the etiology, incidence, and prevalence are missing or significantly limited. More importantly to the individuals living with SCI, health care for SCI patients is not concentrated in specialized SCI centers but fragmented, which might result in a less effective recovery and rehabilitation of patients. Accordingly, the Paracelsus Medical University Salzburg (PMU) and the Austrian Social Insurance for Occupational Risks (AUVA) initiated the Austrian Spinal Cord Injury Study (ASCIS) at the beginning of 2012. ASCIS is defined as an organized network that uses observational methods to collect uniform longitudinal clinical data that can provide insight into current patient care parameters and evaluates the outcome of SCI patients. ASCIS was initiated with the aim to facilitate clinical research and evidence-based practice in care delivery by creating a national registry of individuals, who have sustained an acute tSCI and connecting clinicians, researchers, and patients with SCI. The development and implementation of the ASCIS registry in Austria has grown in strength and has become increasingly important in recent years. Due to the involvement of acute trauma hospitals and rehabilitation centers, the ASCIS is uniquely positioned to capture detailed hospital-related information on the acute, rehabilitation, and chronic phases of tSCI patients. The creation and utilization of databases such as the ASCIS database have increased the reporting of information on the occurrence of SCI in Austria. The collected data elements encompass a broad range of information and uses standard definitions to be compared and shared between centers, countries, and research studies to improve understanding of how to best prevent and treat SCI. ASCIS can provide information and insight into a number of research topics on physical status or disease progression and health care. The collected patient data in the ASCIS registry have the ability to support clinicians with information about which acute care or rehabilitation interventions contribute to best patient outcome, especially for SCI, in which neurologic recovery varies widely. Knowing from international SCI registries such as the European Multi-Center Study about Spinal Cord Injury (EMSCI), which has included the ASCIS as a cooperating partner since the end of 2014, and the Rick Hansen Spinal Cord Injury Registry in Canada [8], large-scale observational studies on topics such as validating the American Spinal Injury Association (ASIA) Impairment Scale (AIS) and examining spontaneous recovery patterns has been already facilitated [8–10]. Additionally, other SCI registries have contributed to our understanding of tSCI epidemiology and quality of life following trauma [11–13]. The purpose of this article is to provide an overview of the development of the ASCIS, the types of data collected, and the scientific research questions ASCIS will address.

## Methods

All patients clinically diagnosed with an acute tSCI are eligible to be included in the database and are approached to obtain informed consent. Clinical scoring was performed according to the International Standards for Neurological Classification of Spinal Injury (ISNCSCI) released by the American Spinal Injury Association (ASIA) [14]. Patients diagnosed with AIS E were excluded in the study. Inclusion criteria are a single event of traumatic para- or tetraplegia, first ASCIS assessment possible in the acute phase (day 0–40) after incidence and patient capable and willing of giving written informed consent. Patients with a non-tSCI (e.g., discus herniation, tumor, AV malformation), severe cognitive impairment (e.g., previously known dementia or severe reduction of intelligence, leading to reduced capabilities of cooperation or giving consent), peripheral nerve lesion above the level of lesion (e.g., plexus brachialis impairment), previously known polyneuropathy or serious traumatic brain injury, and those with reduced ability to cooperate or provide consent are not included. Patient data are collected at each ASCIS site. The central administrative database is managed by the PMU Department of Traumatology and Orthopedics.

All ASCIS sites obtain study approval from the leading ethical board in Salzburg; this process ensures that research is conducted in accordance with the best practices for protecting privacy in health research. We certify that all applicable institutional and governmental regulations concerning the ethical use of patient data of SCI patients were followed during the course of this research. All participating ASCIS sites adhere to one standardized study protocol prepared by the national ASCIS team. Subjects provide written informed consent before enrolling in the study. To ensure protection of patients’ privacy, the data are de-identified when they leave local ASCIS sites. Identification of patient information is maintained between local and national data sets via a unique ASCIS identification number, which links to the personal identifiers associated with patient information at local sites. The registry identification numbers are also used to prevent duplication in data collection and to facilitate the tracking of patient transfers between sites. The registry identification number will be provided from the ASCIS coordinating center for the local sites and will be used once for the patient. This ASCIS registry number will be indicated in the physician’s letter of the patient. In case of transferring the patient to another ASCIS participating hospital, the problem of double registrations will be avoided.

## Results

### ASCIS network

**Fig. 1:**
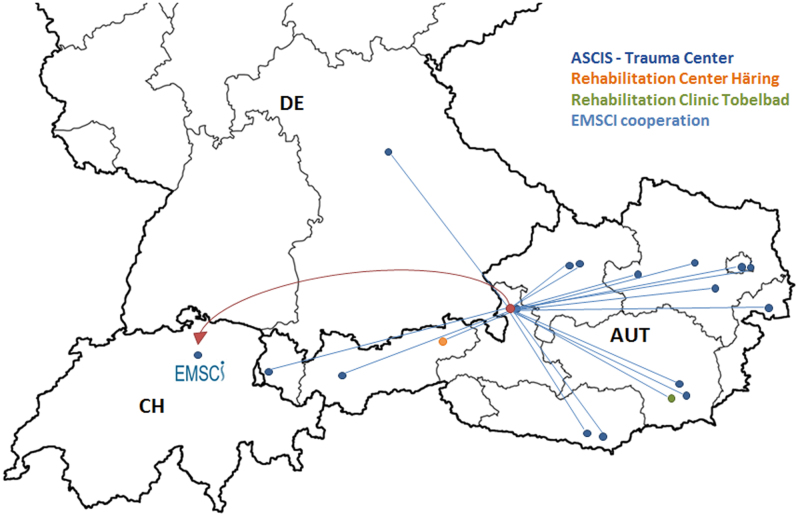
ASCIS network. The ASCIS network comprises 21 participating centers including rehabilitation units, university clinics, emergency hospitals, and level 1 trauma centers. Clinics that have already included patients in the ASCIS are depicted. The ASCIS will provide collected patient data to the EMSCI database semiannually

The ASCIS registry was designed to be multi-centered and to include both acute and rehabilitation units. It aims to obtain a sufficient volume of data for research and to capture data throughout the health-care continuum, from the early acute phase to the chronic phase after injury. The ASCIS network currently consists of 19 trauma centers and includes 2 centers in Germany and 2 of the largest SCI rehabilitation centers in Austria (Fig. 1). The ASCIS has been implemented in 17 Austrian centers in 9 states to date (Fig. 1). Currently, with local sites actively enrolling and collecting data, over 153 unique participants (February 2017) have been included in the ASCIS database. Since the end of 2014, the ASCIS has been listed as a cooperation partner of EMSCI.

### ASCIS assessments and timeline

**Fig. 2:**
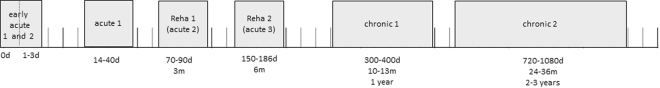
ASCIS schedule. The ASCIS schedule consists of a 7-stage follow-up examination process that collects data from the early acute phase at day 0 until 2–3 years after injury. The data are collected during the early acute phase (between 0 and 1–3 days after injury), the acute/rehabilitation phase (acute 1: 14–40 days after injury; acute 2: 70–90 days after injury; acute 3: 150–86 days after injury), and the chronic phase (chronic 1: 10–13 months after injury; chronic 2: 24–36 months after injury)

For patients who consent to participate in the ASCIS, data elements are collected during the early acute phase and the inpatient rehabilitation phase of their care. The collected data include socio-demographic factors, medical history, injury details, concomitant injuries, diagnoses, interventions, infections, neurologic impairment, complications, functionality, and patient-reported outcomes (Table 1; Supplementary Appendix; summary of ASCIS data elements). These data are collected using a 7-stage follow-up examination; the last evaluation is performed 2–3 years after the injury (Fig. 2). Data recorded by the local ASCIS representatives are based on standard definitions. ASCIS data elements are aligned with International SCI data set forms to ensure that the registry can facilitate the exchange of data internationally [15]. Since the end of 2014, the ASCIS has collaborated with EMSCI and adhered to its schedule, assessments, and international SCI data set forms. During the indicated period, trained physicians perform the ISNCSCI form examinations and subsequent classifications. If patients had not been registered previously, or do not agree in study participation a “minimal dataset” also called “ASCIS registry form” is collected via medical record abstraction. The purpose of this form is to allow ASCIS to document additional newly injured patients treated at their facilities and to enhance epidemiologic investigations by use of the database. This form contains very limited demographic, etiologic, and injury severity information, as well as date of injury in a de-identified manner. No follow-up data collection is performed on these patients. Additionally, this registry form was initiated for patients who do not meet all of the eligibility criteria cited previously, e.g., those with severe traumatic brain injury. In this way, the coverage of the ASCIS register has improved over time.

### ASCIS data- and interface management

**Fig. 3:**
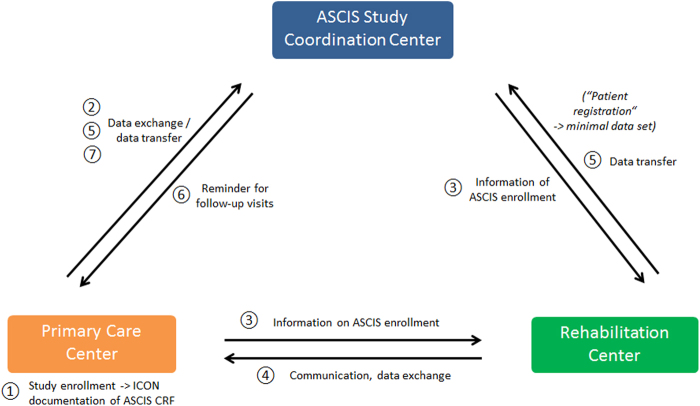
Data and interference management. ➀ Study enrollment will be performed after ICON has been obtained at the PCC. ➁ Data from the acute phase will be forwarded to ASCIS study coordination center. ➂ The PCC will inform the rehabilitation center of ASCIS patients. ➃ ASCIS data from the rehabilitation center will be forwarded either to the PCC (where data elements will be included in the CRF) or ➄ directly to ASCIS study coordination center. ➅ ASCIS study coordination center will remind the PCC about follow-up visits; data from the chronic phase will be forwarded to study coordination center.* CRF* case report form,* ICON* informed consent, *PCC* primary care center

After enrolling the patient in the ASCIS study via signing the informed consent, the data collection begins in the early phase after injury. All data are transferred in a de-identified manner using an ASCIS identification number, which links the personal identifiers associated with patient information at local ASCIS centers. A detailed process of the ASCIS data management will be depicted in Fig. 3 and described more precisely in the text below.

#### Early acute phase/acute phase

➀ The registration process begins in the trauma unit after patient stabilization. After patients enroll in the study, data from the case report form (CRF) or electronic CRF during the acute phase are forwarded to ASCIS study coordinating center ➁ (Fig. 3). All incoming data sets are reviewed and verified for plausibility and completeness to maintain data quality. ➂ The primary care unit informs the proposed rehabilitation center of the patient’s enrollment in the study, and ASCIS study coordination center communicates with the rehabilitation center for ASCIS follow-up visits.

#### Rehabilitation phase

➃ Data collected from the rehabilitation center are either forwarded to the primary care center for further completion of the CRF or are directly transferred to the ASCIS study coordination center. ➄ Assignment of a unique ASCIS patient number enables clear identification of participants.

#### Chronic phase

➅ ASCIS participants are contacted 1 and 2–3 years after their injury to complete patient-reported outcome measures. The ASCIS study coordination center reminds the primary care centers of follow-up visit appointments. ➆ Data from the chronic phase are transferred to ASCIS study coordination center after the last follow-up to complete the data set (Fig. 3).

### ASCIS data-sharing agreement

Importantly, a data-sharing agreement was established between the ASCIS-affiliated facilities and PMU to ensure the protection of privacy. The data-sharing agreement was created by the ASCIS lead investigator and the responsible parties and physicians at each center. It determines the responsibilities of each party at the participating sites with respect to ASCIS and creates an accountability framework for data sharing between the local sites. Because ASCIS operates across various types of facilities (e.g., acute care, rehabilitation), it manages only de-identified patient data, which are entered into the local ASCIS database at the PMU Department of Traumatology and Orthopedics in Salzburg.

### ASCIS governance

ASCIS governance and management are under the auspices of the ASCIS Operation and Management Board. The current management board members consist of the AUVA director, two spinal unit directors, two level 1 trauma center directors, and a data coordinator for ASCIS data. The ASCIS governance structure also includes an ASCIS Scientific Committee, which critically reviews data requests. If the Scientific Committee grants approval, access to and usage of national de-identified data have to be provided from the data coordinator in accordance with the privacy protection framework established by PMU. Furthermore, authorship guidelines have been established for scientific articles that emerge from national ASCIS data and recognize both the contribution of data and scientific input to the research project. However, investigators at each ASCIS site retain full control over access to their own local site data and have the possibility to pursue their own institutional studies. This management structure optimizes the operation and use of ASCIS data; ensures maintenance and development with input from ASCIS stakeholder; and facilitates collaboration between SCI Research Centers for clinical studies as well as the management of trauma units and research staff.

### ASCIS database strengths and limitations

The strength of the ASCIS database is the collection of patient data in the early acute phase after injury, performing two evaluations before day 3 after injury. In contrast to other SCI registries, the strength of ASCIS is the collection of longitudinal follow-up data, performing the last evaluation 2–3 years after injury. Furthermore, ASCIS uses previously established, valid and reliable measures to facilitate comparison with results of other studies. Another strong point of ASCIS is that all incoming data sets are verified at the ASCIS study coordination center for plausibility, completeness, and accuracy to strengthen the quality of the data being reported and maintained in the database. All neurological evaluations using the AIS Grade are controlled using the ISNCSCI form classifier, which is provided by our EMSCI cooperating partner to maintain and improve data quality.

The main limitation of the ASCIS database is that it is not population-based. It includes only persons who were treated in one of the defined ASCIS sites. As some patients with tSCI, particularly those with AIS-D are admitted to community hospitals, the ASCIS does not capture all cases of tSCI in Austria. Therefore, it cannot be used to calculate important epidemiologic measures such as incidence and prevalence. However, the centers with the highest patient volume were selected as ASCIS sites. Another limitation results from missing and incomplete data. Many patients are eventually lost to follow-up as some patients included in the ASCIS database performing their rehabilitation at their home countries. Also, ASCIS does not include non-traumatic SCI patients, as they are often treated in other specialized hospital units rather than at trauma units. Patients younger than 15 years of age are not currently included in the participating spinal units because children with SCI are typically treated at pediatric hospitals. A further limitation of all SCI registries, including ASCIS, is the lack of standardized qualitative and quantitative measures of rehabilitation. To address this gap, we are working on incorporating additional rehabilitation elements based on those already established within the EMSCI network. As several raters may assess one patient over time, inter-rater reliability could remain a problem, even if regular assessment trainings within the ASCIS network are performed.

**Table 1:** A summary of addressed research questions within ASCIS

*General goals for ASCIS:*
To study the longitudinal course of tSCI injury and factors that affect that course
To identify and evaluate trends in etiology, demographic, and injury characteristics of tSCI patients
To identify and evaluate trends over time in health service delivery and treatment outcomes of persons who incur tSCI
To establish expected rehabilitation treatment outcomes for persons with SCI
To facilitate the research and patient care via identifying potential persons for enrollment in appropriate ongoing SCI clinical trials and research projects
Using the ASCIS database for population-based studies
*Examples of specified research questions ASCIS is pursuing:*
Are there differences in the mechanisms of injury and the types of SCI in Austria?
Do elderly patients with a tSCI have better outcomes following surgical or conservative intervention?
Among patients who undergo surgery, does the timing of surgical intervention (e.g., decompression) affect patient outcomes?
Do delays in admission to specialized, acute, and rehabilitation centers increase the incidence of complications, the length of stay or patient outcomes?
How accurate are the ICD-10 diagnostic codes compared with the ISNCSCI data collected by clinicians for describing neurological impairment?
How many centers apply steroid protocols?
How does the quality of life of people with tSCI in Austria compare across provinces? How does it compare with other countries?
Is earlier infection associated with patient outcomes? What types of infections affect the outcome?
Do patients who receive care at a specialized trauma or rehabilitation center have better long-term outcomes than those who are treated at non-specialized centers?
How does functionality influence neurological recovery in terms of different walking parameters?
How does pain influence neurological recovery?
How does concomitant injury influence neurological recovery?

*ICD-10* International Classification of Diseases-10, *ISNCSCI* International Standards for Neurological Classification of SCI, *ISS* Injury Severity Score, *SCI* spinal cord injury

## Discussion

The ASCIS registry has facilitated clinical research through the data collected from current ASCIS sites. An ongoing project using ASCIS data is investigating which patient flow models result in superior patient outcomes as a precursor to creating and implementing new best practices. Implementation of the ASCIS has established standards for SCI clinical practice at participating ASCIS sites and has already positively influenced patient care. For example, during the ASCIS development phase, the aligned data collection in trauma centers and rehabilitation centers led to substantially enhanced communication and continuity across the care continuum. The standardization of data collection at ASCIS sites has ensured that each site received training on administering the ISNCSCI form and the International Core Dataset from qualified and trained clinical staff [14, 15]. This process has not only improved the data quality but has also provided ongoing training for all SCI clinical staff at ASCIS-affiliated facilities. Since the implementation of ASCIS, there has been an increase in the number of ASCIS centers using the ISNCSCI form, thereby improving the clinical reporting of SCI. Furthermore, performing follow-up assessments of ASCIS participants provides information related to their SCI and state of health. ASCIS data are being used to understand how patients move through the Austrian health-care system and the differences in care provided between the states. In the future, ASCIS can be used to determine the feasibility of sites’ participation in clinical trials and to identify individuals with tSCI who are interested in participating in research studies.

The ASCIS database has been designed to track current and longitudinal clinical data for patients with tSCI. It can be used as a powerful tool to observe the course of disease, to understand variations in treatment and outcomes; to examine factors that influence prognosis and quality of life; to describe care patterns including appropriateness of care and disparities in the delivery of care; to measure quality of care and provide an infrastructure for clinical trials. Additionally the database can be used to study quality improvement of care for tSCI patients. Especially the collection of data in the early acute phase of injury in particular makes the ASCIS unique among SCI registries. The ASCIS provides a comprehensive national data set, which may be a useful tool in planning multi-center clinical SCI practices and is another step toward achieving the vision of ensuring the best possible health and well-being of people with tSCI in Austria.

### Electronic supplementary material

ASCIS data elements

## Appendix

**Principle investigators (of active centers) of the ASCIS consortium:**

T. Freude, R. Bogner, M. Blocher, G. Wagenhofer, T. Grossauer from the Department of Orthopaedics and Traumatology, Paracelsus Medical University Salzburg, Salzburg, Austria; E. Trinka, S. Leis from the Department of Neurology, Paracelsus Medical University Salzburg, Salzburg, Austria; A. Gohm, M. Osti, V. Bauer from the Department of Trauma Surgery and Sports Traumatology, Academic Hospital Feldkirch, Vorarlberg, Austria; J. Obrist, M. Lill from the Trauma Center Salzburg, Teaching Hospital of the Paracelsus Medical University Salzburg, Salzburg Austria; O. Kwasny, S. Zohner, M. Klein from the Department of Orthopaedics and Traumatology, Medcampus III, Kepler University Linz, Linz, Upper Austria, Austria; K. Katzensteiner, G. Mattiassich, R. Hafner from the Trauma Center Linz, Teaching Hospital of the Paracelsus Medical University Salzburg, Linz, Upper Austria, Austria; A. Gruber, M. Gollwitzer, A. Grimmer from the Department of Neurosurgery, Neuromed Campus, Kepler University Linz, Linz, Upper Austria, Austria; H. Schnell, A. Wels, M. Kronawetter from the Department of Orthopaedics and Traumatology, University Clinic St. Pölten, St. Pölten, Lower Austria, Austria; G. Pajenda, T. Haider from the University Clinic for Trauma Surgery, Medical University of Vienna, Vienna, Austria; F.J. Seibert, E. Tackner, P. Puchwein from the University Clinic for Trauma Surgery, Medical University of Graz, Graz, Styria, Austria; M. Plecko, G. Kohrgruber, N. Lind from the Trauma Center Graz, Graz, Styria, Austria; E. J. Müller, A. Gstrein, S. Monsberger from the Department of Trauma Surgery and Sports Traumatology Klagenfurt, Klagenfurt, Carinthia, Austria; V. Smekal, R. Pranzl, M. Treven from the Trauma Center Klagenfurt, Klagenfurt, Carinthia, Austria; A. Kathrein, A. Irenberger, S. Kaser from the Trauma hospital St. Vinzenz in Zams, Zams, Tyrol, Austria; H. Boszotta, D. Böckmann from the Department of Orthopaedic Surgery, Ordensklinikum Barmherzige Brüder Eisenstadt, Eisenstadt, Burgenland, Austria; A. Pachucki, R. Burgstaller from the Landesklinikum Amstetten, Amstetten, Lower Austria, Austria; F. Ortner, K. Gruber from the Landesklinikum Wiener Neustadt, Wiener Neustadt, Vienna, Austria; M. Mousavi, S. Rabel from the SMZ-Ost Donauspital, Vienna, Austria; B. Huber, G. Birbamer from the Rehabilitation Center Bad Häring, Bad Häring, Tyrol, Austria; R. Wildburger, G. Wittgruber, A. Grazer-Horacek from the Rehabilitation Clinic Tobelbad, Tobelbad, Styria, Austria; A. Greslehner, W. Schaden, Medical Management from the AUVA.
